# Risk of atrial fibrillation in patients with multiple myeloma: what is known and directions for future study

**DOI:** 10.1186/s43044-023-00434-6

**Published:** 2024-02-01

**Authors:** Ting Fu, Yuxiao Chen, Lian Lou, Zhihang Li, Wen Shi, Xuan Zhang, Jian Yang

**Affiliations:** 1 Department of Cardiology, The First Affiliated Hospital, College of Medicine, Zhejiang University, Hangzhou, China; 2 Department of Cardiology, Yiwu Central Hospital, Jinhua, China

**Keywords:** Multiple myeloma, Atrial fibrillation

## Abstract

**Background:**

Multiple myeloma (MM) is a prevalent hematological tumor, and recent clinical data have highlighted the significance of atrial fibrillation (AF) as a crucial complication affecting the prognosis of MM. This review aims to consolidate findings from published clinical studies, focusing on the epidemiological characteristics of AF in MM patients and the associated risks arising from MM treatments such as autologous hematopoietic stem cell transplantation, proteasome inhibitors, and immunomodulatory agents.

**Main body:**

While existing data partially demonstrate a strong correlation between MM and AF, further clinical studies are necessary to comprehensively investigate their association. These studies should encompass various aspects, including the risk of AF resulting from MM treatment, the impact of AF-induced embolic events and heart failure on MM prognosis, as well as the influence of AF management methods like catheter ablation or left atrial appendage closure on MM prognosis.

**Conclusions:**

The supplementation of future data will provide more precise guidance for managing MM patients. By incorporating information regarding AF risk associated with MM treatment and examining the effects of AF management strategies on MM prognosis, healthcare professionals can enhance their decision-making process when caring for individuals with MM.

## Background

Multiple myeloma (MM) is one of the most common hematological malignancies, accounting for 0.9% of all cancers worldwide in 2020, with 176,404 new cases and 117,077 deaths [1]. The survival of MM has significantly improved with the development of new therapies such as immunomodulatory drugs (IMiDs), proteasome inhibitors (PIs), and autologous hematopoietic stem cell transplantation (ASCT) [2]. Prior to 2000, relapsed MM patients had a reported median survival of 12 months, which increased to 24 months after 2000 [3]. Another study observed an increase in the 5-year relative survival rate of MM patients from 34% in 1989–1992 to 56% in 2001–2005 [4].

However, despite the encouraging progress in treating the primary disease, more than half of real-world MM patients have comorbidities that directly influence clinical decision-making and significantly increase the risk of death [5]. Among these comorbidities, cardiovascular complications are particularly noteworthy [6]. Previous clinical practice has recognized that MM patients are at a higher risk of developing heart failure due to various treatments or secondary cardiac amyloidosis [7]. With advancements in treatment methods and deeper clinical understanding, another common cardiac complication receiving increasing attention is atrial fibrillation (AF). Studies have shown that MM patients with AF face a significantly increased risk of death and medical costs [8]. Furthermore, a recent study published in the *Journal of the American College of Cardiology* reported that MM patients have the highest risk of developing AF compared to other cancer patients [9].

In summary, AF is a comorbidity that cannot be ignored in MM patients. Currently, there is a lack of comprehensive summaries and reviews regarding clinical data on the co-occurrence of MM and AF. The purpose of this review is to summarize existing research, systematically explore the connection between MM and AF, and provide a reference for assessing the risk of AF in MM management.

## Main text

### Epidemiology

Some studies have reported the epidemiological correlation between MM and AF (Table 1).s A study utilizing the National Inpatient Sample (NIS) database, the largest inpatient database in the United States, discovered that among 40,030,380 adult cancer patients diagnosed between 2005 and 2015, the prevalence of AF was 14.6% [10]. When observing MM patients, the prevalence of AF in three age groups was as follows: group 1 (< 65 years old)—22,646/345,247 (6.6%), group 2 (65–80 years old)—84,372/457,525 (18.4%), and group 3 (> 80 years old)—52,551/186,174 (28.2%). Multiple regression analysis demonstrated a significant increase in the risk of AF associated with MM. The odds ratio (OR) values with their corresponding 95% confidence interval (CI) for groups 1, 2, and 3 were as follows: group 1—OR = 1.59 [1.57–1.61], *P* < 0.0001; group 2—OR = 1.21 [1.20–1.22], *P* < 0.0001; group 3—OR = 1.01 [1.00–1.02], *P* = 0.006. Importantly, combined AF significantly elevated mortality rates in MM patients: group 1—(4.3% vs. 2.5%, *P* < 0.0001); group 2—(3.7% vs. 3.3%, *P* < 0.0001).

**Table 1:** Details of the literature with epidemiology

References	Type	Area	Population	Sample size	Conclusion
Yun et al. [9]	Cohort study based on health insurance database	Korea	Patients who were diagnosed with cancer between 2009 and 2016 were enrolled Non-cancer subjects were selected as control group	816,811 patients with cancer and 1,633,663 non-cancer controls were enrolled	During a median follow-up of 4.5 years, cancer was an independent risk factor for incident atrial fibrillation (AF) (adjusted subdistribution hazard ratio [aHR]: 1.63; 95% confidence interval [CI]: 1.61 to 1.66). The impact on AF development varied by cancer type, and multiple myeloma (MM) showed a higher association with incident AF (aHR: 3.34; 95% CI: 2.98 to 3.75)
Khan et al. [10]	Cross-sectional study based on national inpatient database	United States	All adult patients (≥ 18 years) who were hospitalizations and diagnosed with cancer between 2005 to 2015	40,030,380 patients with cancer were included, and the prevalence of AF in these cancer patients was 14.6%	In patients age < 80 years, AF has significant association with MM and lung cancer, where as in patients age > 80 years, it has significant association with non-Hodgkin lymphoma and prostate cancer
Li et al. [11]	Single-center retrospective cohort study	Xi’an, China	Patients with MM between January 2015 to April 2020	A total of 319 patients with MM were included, with 6% combined AF	During a median follow-up of 18.3 months, the all-cause mortality rate of MM patients with AF and without arrhythmias were 73.7% (14/19) and 50.6% (84/166)

Another study conducted using the Korean National Health Insurance Service (NHIS) database, a government-administered insurance plan covering nearly the entire population, included all newly diagnosed cancer patients from January 1st, 2009 to December 31st, 2016. Follow-up was conducted until December 13th, 2017 to observe the risk of AF in cancer patients [9]. The data revealed the following probabilities and incidence rates of new-onset AF within specific time frames of MM diagnosis: within 90 days—probability: 282/4,034 (7.0%), incidence rate: 19.95 per 1000 person-years; within 1 year—probability: 277/3,843 (7.2%), incidence rate: 20.36 per 1000 person-years; within 5 years—probability: 34/1,087 (3.1%), incidence rate: 20.14 per 1000 person-years.

Additionally, a survey conducted at a single center in China reported a prevalence rate of AF in MM patients as 19/319 (6.0%) [11].

### Atrial fibrillation risk during multiple myeloma treatment

#### Autologous hematopoietic stem cell transplantation

ASCT has become one of the preferred treatment options for newly diagnosed MM patients after induction therapy [12]. However, exisiting evidence indicates that cardiotoxicity is a significant complication that cannot be ignored following ASCT [13]. ASCT survivors have a 2–4 times higher risk of cardiovascular death compared to general population [14, 15]. AF is the most common cardiac arrhythmia observed after ASCT [16]. The incidence of AF in MM patients after ASCT has been reported to be as high as 27%, occurring at a mean duration of 14.8 days following the procedure [17].

Multiple regression analysis revealed several factors significantly associated with AF after ASCT: baseline renal dysfunction (OR 15.2 [5.08–45.6]), left ventricular systolic dysfunction (OR 9.55 [2.78–32.79]), dilated left atrium on echocardiogram (OR 4.97 [1.8–13.78]), and hypertension (OR 3.6 [1.36–9.52]) [17]. Moreover, a study conducted at Mayo Clinic identified baseline diastolic dysfunction and weight gain greater than 7% as significant risk factors for AF in MM patients undergoing ASCT [18]. Within this study, researchers found that out of 395 MM patients undergoing ASCT, there were 39 cases of new-onset AF (9.9%) during a median follow-up period of 2.6 years, with approximately 72% occurring within 21 days after the transplantation.

#### Melphalan

ASCT with high-dose melphalan conditioning was once considered the standard therapy for MM [19, 20]. The recommended dosage for melphalan conditioning in patients with normal renal function is 200 mg/m^2^ [21]. In a clinical trial reported in 1999, investigating melphalan induction at a dose of 220 mg/m^2^ combined with ASCT, 2 out of 27 subjects (7.4%) developed paroxysmal AF following melphalan treatment and were successfully treated with amiodarone [22]. Similarly, when investigating a dose of 280 mg/m^2^ of melphalan, 3 out of 36 subjects (8.3%) developed AF [23].

Palifermin is a recombinant human keratinocyte growth factor approved for the prevention of oral mucositis in chemoradiotherapy for hematological malignancies [24, 25]. In a phase I dose-escalation trial of palifermin plus melphalan, among 18 subjects with normal renal function who received up to 280 mg/m^2^ of melphalan, one subject developed AF [26]. Considering that renal failure is associated with increased melphalan toxicity [27], studies recommend reducing the melphalan dose to 140 mg/m^2^ in patients with renal insufficiency [28, 29]. Another study involving MM patients with a creatinine clearance of 60 mL/min/1.73m^2^ or lower found that melphalan at a dose of 180 mg/m^2^ was safe when used in combination with palifermin [30]. Among the 15 subjects, AF was not observed except in one patient who had a history of AF and developed it during dose escalation to 160 mg/m^2^.

Additionally, Mileshkin et al. found in their study that patients aged 60 years or older had an increased risk of cardiotoxicity (particularly AF) compared to younger adults during high-dose chemotherapy and ASCT. However, these complications were manageable and similar to the long-term prognosis of younger adults [31].

#### Lenalidomide

Lenalidomide is an immunomodulatory agent that significantly improves the survival rate of patients with MM and plays a crucial role in both induction therapy and maintenance therapy for MM [12, 32]. While myelosuppression and infection are common adverse effects, it is important to address the increased risk of AF associated with lenalidomide use [33]. In a clinical trial investigating the combination of lenalidomide and dexamethasone for MM treatment, the incidence of AF was 2.9% (10/346) in the “lenalidomide + dexamethasone” group compared to 0.9% (3/345) in the “placebo + dexamethasone” group [34].

#### Proteosome inhibitor

Proteasome inhibitors, such as bortezomib, have been shown to significantly improve the prognosis of patients with MM. A study utilizing the United States Food and Drug Administration (FDA) Adverse Event Reporting System (FAERS) database assessed the risk of AF associated with various anticancer drugs [35]. The findings indicated that bortezomib (1.5%) and carfilzomib (1.5%) ranked second in terms of AF risk, following ibrutinib (5.3%) and venetoclax (1.6%).

#### Daratumumab

Between 2015 and 2020, the United States FDA approved six new drugs for treating the MM: elotuzumab, which activates natural killer cells [36]; ixazomib, a proteasome inhibitor [37]; daratumumab, which targets CD38 [38]; panobinostat, an inhibitor of histone deacetylase (HDACs) [39]; belantamab, an antibody drug conjugate that targets B cell maturation antigen [40]; and selinexor, a selective inhibitor of XPO1 [41]. Daratumumab has been incorporated into first-line regimens for MM treatment in clinical practice [12]. Analysis based on the FAERS database revealed a high incidence of AF in MM patients treated with daratumumab, reaching 6.5% [42]. Additionally, MM patients treated with ixazomib had a 3.5% risk of AF.

#### Histone deacetylase inhibitor

HDACs are expressed at increased levels in various malignant tumors and are considered one of the prominent targets for anticancer therapy [43]. Panobinostat (mentioned earlier) is the first FDA-approved HDAC inhibitor for MM treatment and has shown a 1.3% incidence of AF in MM patients based on data from the FAERS database [42]. Quisinostat, a highly potent second-generation selective HDAC inhibitor with orally activity, has demonstrated promising therapeutic effects against MM in rodent models and human samples tested in vitro [44–46]. Considering the synergistic effect observed between quisinostat and bortezomib in mice, their combined regimen is currently under further investigation [47]. In a phase Ib dose study involving 12 mg of quisinostat, AF was reported in 1 out of 6 subjects (16.7%) [48].

## Conclusions

MM is a prevalent hematological malignancy that has witnessed significant advancements in treatment methods leading to improved prognosis. Consequently, managing complications has emerged as a critical approach for optimizing MM treatment further. This review aims to provide a comprehensive analysis by aggregating relevant data from clinical trials and observational studies to elucidate the association between MM and AF. Existing evidence suggests that MM may have the strongest correlation with AF among all cancers. The prevalence of AF in patients with MM is notably high and increases with age, reaching nearly 30% [10]. Moreover, combined treatments for MM often elevate the risk of AF. We have discussed the risks of AF associated with ASCT, melphalan, lenalidomide, daratumumab, and other therapeutic modalities. However, several commonly used treatments lack sufficient observational data regarding their impact of AF risk.

Cancer is characterized by changes in inflammation levels [49–51], which also serve as a significant risk factor for AF [52–54]. This theory partially explains the association between MM and AF. Notably, research has demonstrated an association between AF and almost all cancer subtypes, suggesting an independent link between cancer and AF [55]. The emerging field of onco-cardiology emphasizes the importance of managing cardiovascular complications to improve cancer prognosis. In cancers requiring systemic therapy such as chemotherapy or immunotherapy, cardiovascular complications exert a more substantial influence on prognosis. Given MM patients’ heightened risk of AF, it is crucial to explore further how these two conditions interact and impact individual prognoses.

The current clinical data on the relationship between MM and AF are overall very limited. Many newly introduced drugs in clinical practice still lack comprehensive observational data regarding their impact on MM and AF, including immunomodulatory agents, proteasome inhibitors, and HDAC inhibitors. However, studies conducted by Al-Yafeai et al. and Ahmad et al. partially address this knowledge gap [35, 42]. Despite the available data, the risk of AF is often incidentally reported and underappreciated by researchers. It is important to note that although “cardiovascular disease” is a general term used in complication reports of clinical research articles, it encompasses various conditions such as coronary heart disease, heart failure, arrhythmia, and cardiomyopathy that differ significantly in management and prognosis. Therefore, more detailed classification and reporting, similar to high-quality research reports, are necessary.

The risk of AF at each stage of MM management necessitates careful consideration by clinicians regarding its advantages and disadvantages. However, there is a scarcity of evidence available for reference. Despite limited observational studies reporting on the likelihood of developing AF with specific treatment modalities, clinicians often struggle to effectively compare the risks associated with AF against potential clinical benefits, leading to challenges in making rational decisions. The primary risks associated with AF are embolic events and heart failure, both potentially fatal. However, in MM patients, there is lack of in-depth clinical data to elucidate the correlations between AF and these risks. Therefore, dedicated clinical investigations are required to comprehensively analyze the risk and prognosis of AF in MM-specific drug treatments. Moreover, it remains unclear whether aggressive treatment for AF improves outcomes in MM patients who also have AF. While management approaches like catheter ablation and left atrial appendage occlusion have proven effective in enhancing the prognosis of typical AF patients [56], the perioperative risks of embolic events and bleeding, as well as treatment benefits, need to be evaluated specifically in future clinical trials involving MM patients.

Recent studies have attempted to answer the benefits of AF ablation in cancer patients. Analysis based on the United States National Readmissions Database identified the association between active cancer and higher odds of periprocedural complications and all-cause and bleeding-related readmissions in patients undergoing AF ablation [57]. A META analysis showed that cancer survivors have an increased risk of bleeding after ablation for AF to patients without cancer, with on significant difference in the efficacy of ablation for maintenance of sinus rhythm [58]. The impact of cancer activity on ablation timing, and the impact of ablation of long-term prognosis need to be further demonstrated in future large-scale trials.

## Data Availability

Not applicable.

## Abbreviations

MM: Multiple myeloma
IMiDs: Immunomodulatory drugs
PIs: Proteasome inhibitors
ASCT: Autologous hematopoietic stem cell transplantation
AF: Atrial fibrillation
NIS: National Inpatient Sample
OR: Odds ratio
CI: Confidence interval
NHIS: National Health Insurance Service
FDA: Food and Drug Administration
FAERS: Food and Drug Administration Adverse Event Reporting System
HDACs: Histone deacetylase
